# Effects of occupational exposure to metal fume PM_2.5_ on lung function and biomarkers among shipyard workers: a 3-year prospective cohort study

**DOI:** 10.1007/s00420-024-02055-1

**Published:** 2024-03-13

**Authors:** Huan Minh Tran, Ching-Huang Lai, Wei-Liang Chen, Chung Ching Wang, Che-Wei Liang, Chi-Yu Chien, Chih-Hong Pan, Kai-Jen Chuang, Hsiao-Chi Chuang

**Affiliations:** 1 Program in Global Health and Health Security, College of Public Health, Taipei Medical University, Taipei, Taiwan; 2 Faculty of Public Health, Da Nang University of Medical Technology and Pharmacy, Da Nang, Vietnam; 3 School of Public Health, National Defense Medical Center, Taipei, Taiwan; 4 Division of Family Medicine, Department of Family and Community Medicine, Tri-Service General Hospital, Taipei, Taiwan; 5 Division of Geriatric Medicine, Department of Family and Community Medicine, Tri-Service General Hospital, Taipei, Taiwan; 6 School of Medicine, National Defense Medical Center, Taipei, Taiwan; 7 Institute of Labor, Occupational Safety and Health, Ministry of Labor, New Taipei City, Taiwan; 8 School of Public Health, College of Public Health, Taipei Medical University, Taipei, Taiwan; 9 Department of Public Health, School of Medicine, College of Medicine, Taipei Medical University, Taipei, Taiwan; 10 Division of Pulmonary Medicine, Department of Internal Medicine, Shuang Ho Hospital, Taipei Medical University, New Taipei City, Taiwan; 11 Inhalation Toxicology Research Lab (ITRL), School of Respiratory Therapy, College of Medicine, Taipei Medical University, 250 Wuxing Street, Taipei, 11031 Taiwan; 12 Cell Physiology and Molecular Image Research Center, Wan Fang Hospital, Taipei Medical University, Taipei, Taiwan

**Keywords:** α1-antitrypsin, ITIH4, Lung function, Oxidative stress, Particulate matter, Welding

## Abstract

**Objective:**

This study investigates the associations of α1-antitrypsin, inter-α-trypsin inhibitor heavy chain (ITIH4), and 8-isoprostane with lung function in shipyard workers exposed to occupational metal fume fine particulate matter (PM_2.5_), which is known to be associated with adverse respiratory outcomes.

**Methods:**

A 3-year follow-up study was conducted on 180 shipyard workers with 262 measurements. Personal exposure to welding fume PM_2.5_ was collected for an 8-h working day. Pre-exposure, post-exposure, and delta (∆) levels of α1-antitrypsin, ITIH4, and 8-isoprostane were determined in urine using enzyme-linked immunosorbent assays. Post-exposure urinary metals were sampled at the beginning of the next working day and analyzed by inductively coupled plasma-mass spectrometry. Lung function measurements were also conducted the next working day for post-exposure.

**Results:**

An IQR increase in PM_2.5_ was associated with decreases of 2.157% in FEV_1_, 2.806% in PEF, 4.328% in FEF_25%_, 5.047% in FEF_50%_, and 7.205% in FEF_75%_. An IQR increase in PM_2.5_ led to increases of 42.155 µg/g in ∆α1-antitrypsin and 16.273 µg/g in ∆ITIH4. Notably, IQR increases in various urinary metals were associated with increases in specific biomarkers, such as post-urinary α1-antitrypsin and ITIH4. Moreover, increases in ∆ α1-antitrypsin and ∆ITIH4 were associated with decreases in FEV_1_/FVC by 0.008% and 0.020%, respectively, and an increase in ∆8-isoprostane resulted in a 1.538% decline in FVC.

**Conclusion:**

Our study suggests that urinary α1-antitrypsin and ITIH4 could indicate early lung function decline in shipyard workers exposed to metal fume PM_2.5_, underscoring the need for better safety and health monitoring to reduce respiratory risks.

**Graphical abstract:**

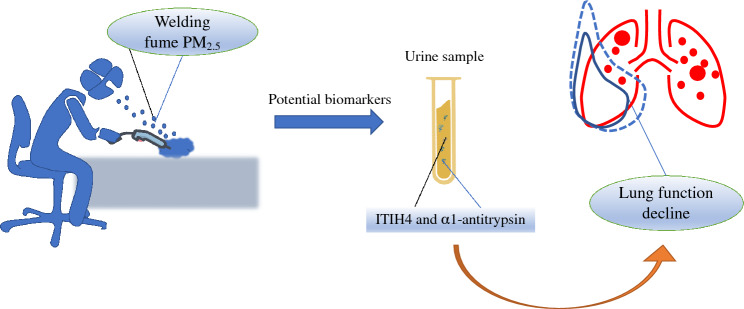

**Supplementary Information:**

The online version contains supplementary material available at 10.1007/s00420-024-02055-1.

## Introduction

Long-term exposure to fine particulate matter with an aerodynamic diameter of less than 2.5 µm (PM_2.5_) has been well-documented for its role in adverse respiratory outcomes, such as declines in lung function and biochemical alterations in the airways (Valavanidis et al. 2008). Previous studies have quantified the risk; a 1 µg/m^3^ increase in PM_2.5_ over various time lags led to a marked decline in forced expiratory volume in 1 s (FEV_1_) among COPD patients (Tran et al. 2022). Given its specific physiological impacts and its heightened risk in occupational settings, PM_2.5_ warrants particular attention over other types of air pollutants like PM_10_. Further complicating the issue is the hazardous composition of PM_2.5_, which contributes to respiratory outcomes (Antonini et al. 2004).

In occupational settings, high concentrations of hazardous PM_2.5_ have been shown to accelerate declines in lung function, contributing to airflow-limited diseases (Neophytou et al. 2019). The European Community Respiratory Health Survey, focusing on occupational exposure, has shown that a minimum of 15% of cases of asthma and COPD can be attributed to the workplace. Additionally, it was estimated that over 20% of COPD incidences are linked to exposure to occupational dust (Lytras et al. 2018). Another cross-sectional study on metal-containing PM_2.5_ is of particular concern due to its chemical reactivity upon inhalation (Zeng et al. 2016). Diseases with airflow limitations, such as occupational asthma and COPD, have been linked to metals like total chromium (Cr), iron (Fe), lead (Pb), manganese (Mn), and nickel (Ni) (Wang et al. 2022). The decline in lung function would serve as an early indicator for chronic respiratory diseases, especially COPD (Tantucci and Modina 2012).

Shipyards present an occupational environment fraught with elevated risk factors, primarily because of metal-containing PM_2.5_ in welding fumes (Chuang et al. 2018; Lai et al. 2021). Metals such as chromium (Cr), iron (Fe), lead (Pb), manganese (Mn), and nickel (Ni) have been identified in shipyard environments and are implicated in inducing inflammation and oxidative stress in the respiratory system (Niu et al. 2010). These metal fumes not only persist in the lungs but also are detectable in urinary metal assessments, serving as indicators of occupational exposure (Riccelli et al. 2020).

Against this background of occupational PM_2.5_ exposure, identifying specific biomarkers for lung function decline becomes imperative (Riccelli et al. 2020). The α1-antitrypsin, inter-α-trypsin inhibitor heavy chain (ITIH4), and 8-isoprostane are among the biomarkers examined in relation to lung oxidative stress and inflammation (Cazzola et al. 2020; Chen et al. 2021). In particular, α1-antitrypsin has been linked to declines in lung function and worse systemic inflammation among COPD patients (Jonigk et al. 2013). ITIH4 plays a role in inflammation caused by air pollution (Chen et al. 2021). High concentrations of 8-isoprostane might indicate irritative effects in the airways due to exposure to specific metals like Fe and Ni (Hoffmeyer et al. 2012a). A significant gap in existing research is the nuanced understanding of the longitudinal impacts of occupational exposure to metal-containing PM_2.5_ on respiratory health in shipyard workers, particularly the intricate relationships between such exposure, variations in specific biomarkers like α1-antitrypsin, ITIH4, and 8-isoprostane, and the ensuing health outcomes.

**Fig. 1:**
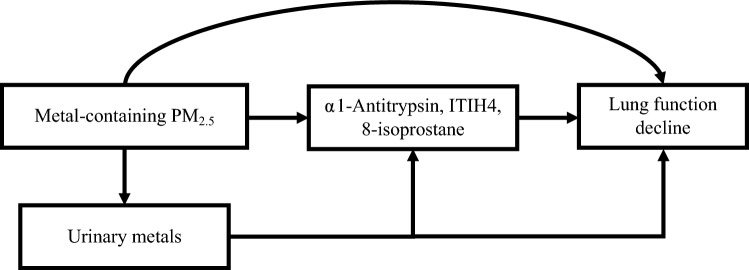
Hypothesized relationships between occupational exposure to metal-containing PM_2.5_, biomarkers, and lung function decline

We hypothesized that shipyard workers exposed to welding fume PM_2.5_ will exhibit a decline in lung function, which can be quantitatively assessed through the elevation of specific biomarkers, namely α1-antitrypsin, ITIH4, and 8-isoprostane (Fig. 1). To validate this hypothesis, our study, utilizing a dynamic cohort design, aimed to explore in depth the relationships between these biomarkers and the deterioration of lung function in the backdrop of metal-containing PM_2.5_ exposure in shipyards. Simultaneously, we assessed the impact of welding fume PM_2.5_ and urinary metals on this decline in lung function. Finally, the links between environmental exposure and lung health would offer valuable insights for occupational safety measures. To validate this hypothesis, our study, utilizing a longitudinal design, aimed to explore in depth the relationships between these biomarkers and the deterioration of lung function in the backdrop of metal-containing PM_2.5_ exposure in shipyards. Simultaneously, we assessed the impact of welding fume PM_2.5_ and urinary metals on this decline in lung function. Finally, the links between environmental exposure and lung health would offer valuable insights for occupational safety measures.

## Materials and methods

### Study population

**Fig. 2:**
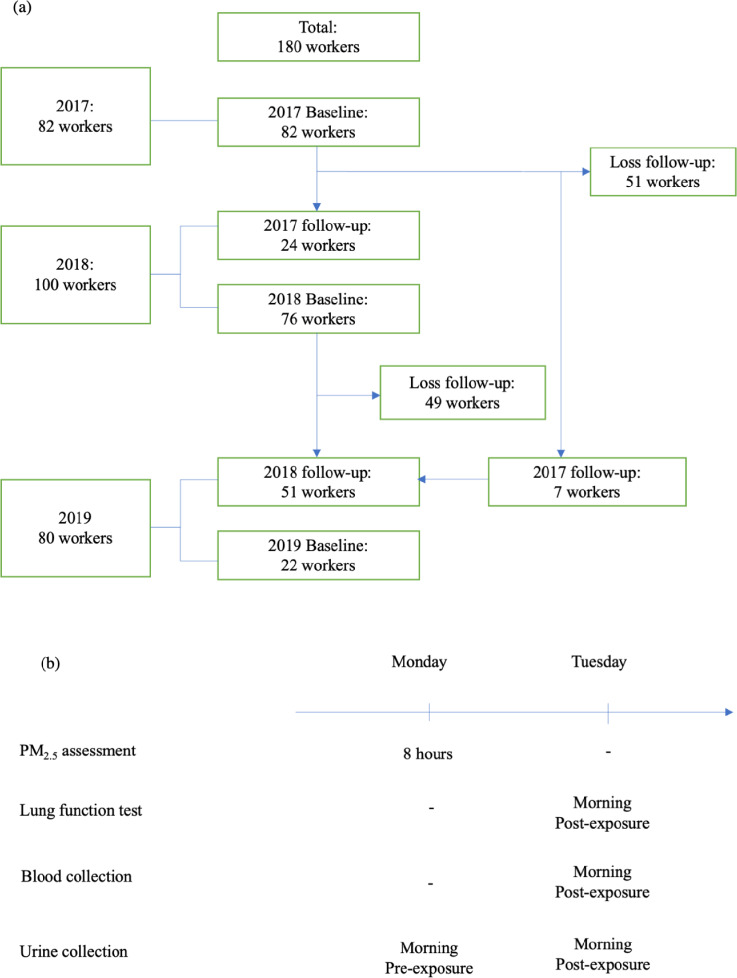
**a** Flowchart of the study of biomarkers for lung function declines due to exposure to metal fume particular matter with an aerodynamic diameter of < 2.5 µm (PM_2.5_) in 3 years of follow-up of shipyard workers (n = 262). **b** Illustration of the experimental procedures for personal PM_2.5_ assessment, lung function, and biomarker collection

A cohort study was conducted between 2017 and 2019 in a northern Taiwan shipyard, focusing on 180 workers involved in tungsten inert gas welding, a dominant welding method at the company (Chuang et al. 2018) (Fig. 2a). Over three visits, we accumulated a total of 262 measurements. On the first visit, 82 subjects were enrolled in the study. On the second visit, 76 subjects were enrolled, 24 subjects were followed-up, but 51 subjects from the first visit withdrew. On the study's third visit, 22 subjects were enrolled, 51 subjects were followed-up, and seven of 51 subjects returned after withdrawing in 2018. We employed a non-random sampling strategy based on voluntary participation but aimed to achieve a representative sample. We included individuals aged between 20 and 70 years to concentrate on those actively participating in shipyard work and to minimize the confounding effects of age-related health issues. We excluded participants who had experienced acute health exacerbations in the month preceding the study to eliminate the influence of recent acute health events on the data. Additionally, individuals diagnosed with specific pulmonary conditions, including tuberculosis, pulmonary infections, and lung cancers, as well as those with cardiovascular diseases and diabetes, were excluded. The low incidence of tuberculosis in Taiwan (State of Health 2023) highlights the rigor of our pulmonary condition exclusion criteria. Although our previous study identified a 12.6% prevalence of COPD among welding workers, none of the participants in the current study were diagnosed with COPD, ensuring that our findings focus specifically on the impacts of occupational exposures.

### Experimental design

Figure 2b outlines the study's experimental procedures. On Monday mornings, each worker was equipped with a personal PM_2.5_ sampler for an 8-h exposure assessment, aligning with a typical workday. The timing for urine sample collection was carefully chosen to capture potential biomarker shifts due to workplace exposure: one sample was taken at the beginning of the workday (08:00 Monday morning; pre-exposure) and another at the start of the following workday (08:00 Tuesday morning; post-exposure). In contrast, plasma samples were only collected post-exposure, at the start of the following workday (Tuesday morning), to capture physiological responses that require a longer time frame to manifest compared to urinary biomarkers. All urine and plasma samples were stored at -80 °C until analysis. A lung function test was measured in each subject on Tuesday morning (post-exposure) during the study periods. Workers' baseline characteristics were gathered through a questionnaire assessing age, sex, BMI, smoking status, alcohol consumption, and dust mask usage. The rationale behind the chosen timing and methods was to create a comprehensive snapshot of both immediate and delayed physiological responses to welding exposure, thus enabling a robust evaluation of the associated health risks.

### Personal PM_2.5_ assessment

The procedure for the personal PM_2.5_ assessment was reported previously (Chuang et al. 2018). A model 200 Personal Environmental Monitor (PEM) equipped with an AirChek® XR5000 air sampling pump (SKC, Eighty-Four, PA, USA) was used to consecutively sample PM_2.5_ for 8 h (09:00 ~ 17:00, Monday) at a flow rate of 2 L/min. The sampler was used to collect PM_2.5_ onto a Teflon filter substrate (37 mm, pore size: 2-m, Pall, Ann Arbor, MI, USA) in the breathing zone. Sampling procedures and quality control procedures were performed according to guidelines of the United States Environmental Protection Agency (USEPA) method IP-10A.

### Lung function

The lung function test was performed using a Chestgraph (Chest, HI-701, Japan) according to American Thoracic Society/European Respiratory Society guidelines (Miller et al. 2005). Forced vital capacity (FVC), FEV_1_, FEV_1_/FVC ratio, peak expiratory flow (PEF), forced expiratory flow at 25% of the FVC (FEF_25%_), forced expiratory flow at 50% of the FVC (FEF_50%_), and forced expiratory flow at 75% of the FVC (FEF_75%_) were measured in each subject on Tuesday morning (post-exposure) during the study periods. To ensure accurate and consistent measurements, the equipment underwent routine calibration and maintenance checks as per manufacturer guidelines.

### Urinary and plasma biomarkers

Plasma and urinary levels of α1-antitrypsin, ITIH4, and 8-isoprostane were quantified using enzyme-linked immunosorbent assays (ELISAs) from R&D Systems and Cayman. Standard curves were constructed for each biomarker based on serial dilutions of known concentrations, and quality control samples were interspersed within each assay plate to assess reproducibility and accuracy. Experiments adhered to the manufacturer-provided guidelines, and all assays were conducted in duplicate. Urinary biomarker concentrations were adjusted for urinary creatinine (µg/g) to account for variations in urinary output.

### Urinary metal concentrations

Urinary metal analyses were conducted using inductively coupled plasma-mass spectrometry (ICP-MS; Agilent 7500, CA, USA) according to a previous study (Chuang et al. 2015). Urine samples were digested with nitric acid using a MARS 5 microwave (CEM, NC, USA). In total, nine metals were selected for analysis, including Cr, Mn, cobalt (Co), Ni, zinc (Zn), cadmium (Cd), copper (Cu), iron (Fe), and vanadium (V), with median coefficients of variation within 5–10%. Analyses were conducted for each target metal when the calibration curve was at its lowest concentration. The method detection limit was determined by multiplying the standard deviation (SD) by three times the magnitude of each urinary metal. Limits of detection (LODs) were 0.005, 0.0018, 0.0003, 0.0108, 0.0679, 0.0007, 0.007, 0.0886, and 0.001 ppb for Cr, Mn, Co, Ni, Zn, Cd, Cu, Fe, and V, respectively. Values below the LOD were replaced by $\frac{LOD}{\sqrt{2}}$. Urinary metal values were normalized by urinary creatinine (µg/g) (Table S1). All urinary metal analyses were performed on post-exposure samples to better evaluate occupational exposure.

### Statistical analysis

The winsorization approach was used to minimize the effects of severe outliers outside the 10th and 90th percentiles (Tsai et al. 2012). Then, normality of the dependent variables was assessed using the Shapiro–Wilk test due to its effectiveness in smaller sample sizes. A paired *t*-test was used to assess changes in different urinary biomarker levels due to exposure to PM_2.5_. Delta (∆) urine levels of biomarkers were defined as post-exposure minus pre-exposure urinary levels. A correlation analysis was conducted using Pearson's correlation coefficients to determine relationships among PM_2.5_, urinary metals, α1-antitrypsin, ITIH4, and 8-isoprostane. Linear mixed-effect models adjusted for age, smoking, and welding duration were used to determine associations of independent variables with dependent variables as follows: (1) PM_2.5_ with plasma, ∆ urine, and post-urinary biomarkers (α1-antitrypsin, ITIH4, and 8-isoprostane), and lung function, (2) urinary metals with biomarkers and lung function, and (3) biomarkers with lung function. Regarding the unbalanced repeated measures in our data, we applied a linear mixed-effects model. The model incorporates both fixed effects—such as age, smoking status, and duration of welding exposure—and random effects, specifically the participant ID codes, to adjust for within-subject correlations across different time points. Age, smoking, and welding duration were selected based on existing literature that supports their impact on the health, especially lung function, of welders (Venkatesan 2023; Yawn et al. 2021). The assumptions of the model, including linearity, independence, and homoscedasticity, were verified prior to the analyses. All of the statistical analyses in this study were conducted with R software (vers. 4.2.1 for macOS). The *plm* package was used for unbalanced panel data analysis (Croissant and Millo 2008). All statistical analyses were conducted at a significance level of *p* < 0.05.

## Results

### Characterization of study subjects

There were 180 subjects enrolled in the study accounting for 262 person-years during the 3 years of follow-up (Table 1). Respective proportions of office workers and welding workers were 28.9% and 71.1%. During the three visits, mean BMI values ranged 24.5–25.7 kg/m^2^. The mean ages of subjects ranged 41.4–47.6 years, while welding durations ranged 4.1–11.6 years. The proportion of subjects that smoked, drank, and always wore a dust mask ranged 28.0–31.8%, 18.2–29.3%, and 35.3–54.5%, respectively.

**Table 1:** Characteristics of subjects at entry-point

Characteristics	1st visit	2nd visit	3rd visit	Total
Number of subjects	82	76	22	180
Person-years	82	100	80	262
Age(years) ± SD	47.6 ± 12.6	41.4 ± 12.5	45.9 ± 12.1	44.2 ± 12.9
BMI,(kg/m^2^) ± SD	25.7 ± 3.5	25.5 ± 3.8	24.5 ± 2.9	25.45 ± 3.55
*Working type, %*				
Office workers	35.4	25.0	18.2	28.9
Welding workers	64.6	75.0	81.8	71.1
*Smoking* (%)				
No smoking	46.4	48.7	54.6	21.7
Ex-smokers	25.6	19.7	13.6	30
Current smokers	28.0	31.6	31.8	48.3
*Drinking alcohol (%)*				
No	70.7	72.4	81.8	72.8
Yes	29.3	27.6	18.2	27.2
Welding duration(years) ± SD	9.0 ± 15.0	4.1 ± 7.2	11.6 ± 15.8	7.23 ± 12.71
*Wearing dust mask (%)*				
Never use	24.4	17.1	13.6	20.0
Sometime	23.2	23.7	9.1	21.7
50% of time	3.7	5.3	4.5	4.4
Most of time	13.4	13.1	18.3	13.9
Always	35.3	40.8	54.5	40.0

*BMI* body-mass index, *SD* standard deviation

### Characterization of PM_2.5_ exposure, lung function, biomarkers, and urinary metals

Personal exposure to PM_2.5_, lung function, and biomarkers, including α1-antitrypsin, ITIH4, and 8-isoprostane levels, were determined in this study (Table 2). During the three visits, we observed that the mean PM_2.5_ exposure ranged 309.6–464.0 µg/m^3^. The means of predicted FVC, FEV_1_, FEV_1_/FVC, PEF, FEF_25%_, FEF_50%_, and FEF_75%_ respectively ranged 93.1–102.4%, 92.8–101.2%, 81.9–84.2%, 88.3–93.7%, 88.8–96.9%, 91.4–98.8%, and 98.6–109.1%. For plasma biomarkers, α1-antitrypsin, ITIH4, and 8-isoprostane levels respectively ranged 276.8–311.9, 87.8–113.2, and 0.04–0.11 µg/L. Post-exposure urinary biomarker levels were significantly lower than pre-exposure levels. Pre- and post-exposure urinary biomarkers levels were determined for α1-antitrypsin (170.9–204.5 and 25.0–43.4 µg/g), ITIH4 (59.3–72.8 and 6.1–10.5 µg/g), and 8-isoprostane (1.22–1.55 and 0.47–0.71 µg/g), respectively. In terms of urinary metals, Cr, Mn, Co, Ni, Zn, Cd, Cu, Fe, and V respectively ranged 4.43–11.78, 1.38–3.43, 0.32–0.34, 6.99–10.6, 513.6–688.2, 0.34–0.79, 22.6–72.6, 74.1–95.6, and 0.36–0.57 µg/g. The distributions of PM_2.5_, 3 biomarkers and urinary metals among shipyard workers were presented in Figure S1 and Figure S2. The correlations of 3 biomarkers and 9 urinary metals were presented in Figure S3 and Figure S4.

**Table 2:** Personal exposure to particular matter with an aerodynamic diameter of < 2.5 µm (PM_2.5_), lung function, levels of biomarkers and metals in urine between pre- and post-exposure in the 1st, 2nd, and 3rd visit in the shipyard

	1st visit		2nd visit		3rd visit	
	Pre-exposure	Post-exposure	Pre-exposure	Post-exposure	Pre-exposure	Post-exposure
PM_2.5_, µg/m^3^(IQR)	464.0 ± 428.8(696.9)		455.6 ± 400.5(669.5)		309.6 ± 421.3(281.5)	
*Lung function (%)*						
FVC	–	95.2 ± 10.6	–	93.1 ± 10.9	–	102.4 ± 8.9
FEV_1_	–	95.5 ± 10.7	–	92.8 ± 11.8	–	101.2 ± 9.7
FEV_1_/FVC	–	81.9 ± 6.1	–	82.8 ± 7.4	–	84.2 ± 7.4
PEF	–	93.7 ± 9.9	–	88.3 ± 14.5	–	93.5 ± 11.2
FEF_25%_	–	96.5 ± 10.7	–	88.8 ± 13.2	–	96.9 ± 10.2
FEF_50%_	–	95.7 ± 12.5	–	91.4 ± 13.9	–	98.8 ± 12.8
FEF_75%_	–	100.5 ± 27.6	–	98.6 ± 25.8	–	109.1 ± 27.2
*α1-antitrypsin*						
In plasma(µg/L)	–	311.9 ± 30.4	–	280.2 ± 38.7	–	276.8 ± 39.5
In urine^b^(µg/g)	171.9 ± 183.8	43.4 ± 34.2^a^	170.9 ± 180.3	29.4 ± 30.5^a^	204.5 ± 205.1	25.0 ± 27.1^a^
*ITIH4*						
In plasma(µg/L)	–	113.2 ± 80.5		87.8 ± 64.7		98.5 ± 56.0
In urine^b^(µg/g)	59.3 ± 57.1	10.5 ± 9.8^a^	63.6 ± 65.9	7.9 ± 8.8^a^	72.8 ± 69.4	6.1 ± 7.8^a^
*8-isoprostane*						
In plasma(µg/L)	–	0.04 ± 0.02	–	0.05 ± 0.03	–	0.11 ± 0.06
In urine^b^(µg/g)	1.22 ± 1.05	0.71 ± 0.47^a^	1.47 ± 1.13	0.54 ± 0.43^a^	1.55 ± 1.07	0.47 ± 0.39^a^
*Urinary metals, µg/g CRE*^*b*^*(IQR)*						
Cr	–	4.43 ± 5.22(3.44)		7.63 ± 8.44(12.80)		11.78 ± 7.10(11.00)
Mn	–	3.43 ± 1.98(2.93)		1.38 ± 1.32(0.97)		3.04 ± 2.06(2.74)
Co	–	0.33 ± 0.28(0.40)		0.32 ± 0.26(0.30)		0.34 ± 0.24(0.24)
Ni	–	10.65 ± 9.15(19.8)		6.99 ± 5.45(5.40)		9.17 ± 6.55(6.13)
Zn	–	575.8 ± 260.2(321.0)		513.6 ± 341.6(493.0)		688.2 ± 270.8(381.4)
Cd	–	0.34 ± 0.40(0.50)		0.69 ± 0.46(0.77)		0.79 ± 0.41(0.59)
Cu	–	72.6 ± 30.0(50.6)		22.6 ± 17.7(17.9)		59.8 ± 32.5(51.2)
Fe	–	75.4 ± 52.2(52.8)		74.1 ± 66.0(76.4)		95.6 ± 72.9(92.5)
V	–	0.39 ± 0.23(0.33)		0.36 ± 0.22(0.28)		0.57 ± 0.26(0.55)

^a^The significant difference between post- and pre-exposure (*p* < 0.05)

^b^The levels of urinary biomarkers and metals were normalized by creatinine

### Associations of PM_2.5_ with lung function parameters and biomarkers

**Fig. 3:**
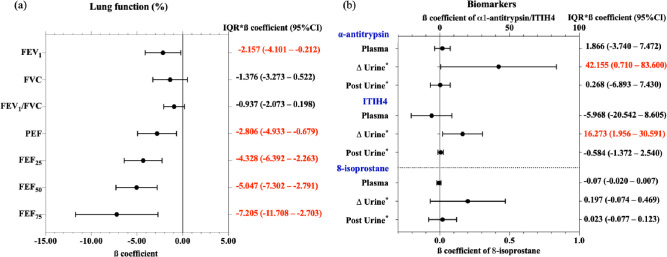
Associations of particulate matter with an aerodynamic diameter of < 2.5 µm (PM_2.5_) with lung function and biomarkers in shipyard workers (n = 262). Delta (∆) exposure is the difference in concentrations of post-exposure and pre-exposure biomarkers. Values with an asterisk (*) were normalized to creatinine. Values in red were deemed to be statistically significant (*p* < 0.05). Covariates adjusted for the models were age, smoking status, and welding duration

Figure 3 demonstrates associations of PM_2.5_ with lung function and biomarkers among study subjects. We found that an interquartile range (IQR) increase in PM_2.5_ was linked to significant decreases in FEV_1_ by 2.157% (95% CI 0.212–4.101%, *p* < 0.05), PEF by 2.806% (95% CI 0.679–4.933%, *p* < 0.05), FEF_25%_ by 4.328% (95% CI 2.263–6.392%, *p* < 0.05), FEF_50%_ by 5.047% (95% CI 2.791–7.302%, *p* < 0.05), and FEF_75%_ by 7.205% (95% CI 2.703–11.718%, *p* < 0.05). Concurrently, the same IQR increase in PM_2.5_ was associated with a rise of 42.155 µg/g in ∆ α1-antitrypsin (95% CI 0.710–83.600, *p* < 0.05) and 16.273 µg/g in ∆ ITIH4 (95% CI 1.956–30.591, *p* < 0.05).

### Associations of urinary metals with biomarkers

**Fig. 4:**
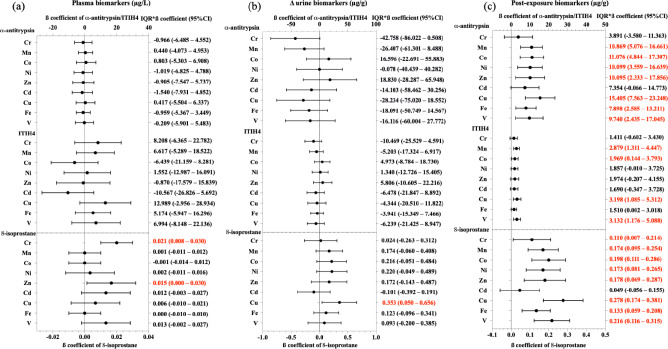
Associations of post-exposure urinary metals after adjusting for urinary creatinine with biomarkers in shipyard workers (n = 262). Delta (∆) urine is the difference in concentrations of post-exposure and pre-exposure urinary biomarkers. ∆ and post-exposure urinary biomarkers were normalized to creatinine. Values in red were deemed to be statistically significant (*p* < 0.05). Covariates adjusted for the models were age, smoking status, and welding duration

Figure 4 demonstrates the associations between urinary metals and specific biomarkers among participants. Notably, IQR increases in Cr and Zn corresponded with rises in plasma 8-isoprostane by 0.021 µg/L and 0.015 µg/L, respectively. In contrast, an IQR elevation in Cu indicated a decrease of 0.353 µg/g in ∆ 8-isoprostane. Furthermore, IQR increases in various metals including Mn, Co, Ni, Zn, Cd, Cu, Fe, and V were associated with significant increases in post urinary α1-antitrypsin, with values ranging from 7.898 µg/g for Cu to 15.405 µg/g for Cd. Additionally, specific IQR increases in Mn, Co, Cu, and V were associated with increases in post urinary ITIH4, ranging from 1.969 µg/g for Co to 3.198 µg/g for Cu. Lastly, increases in Cr, Mn, Co, Ni, Zn, Cu, Fe, and V were associated with varying increases in post urinary 8-isoprostane, from 0.110 µg/g in Cr to 0.278 µg/g in Cu. We also observed that the urinary Cu was negatively associated with FEV_1_, FVC, FEF_50%_, and FEF_75%_ (Figure S5).

### Associations of biomarkers with lung function parameters

**Fig. 5:**
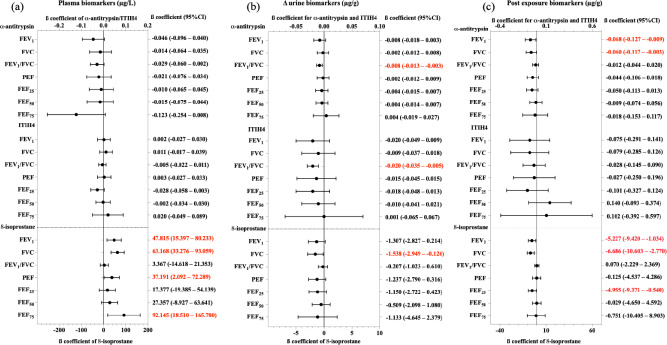
Associations of biomarkers with lung function in shipyard workers. Delta (∆) urine is the difference in concentrations of post-exposure and pre-exposure urinary biomarkers. ∆ urinary and post-exposure urinary biomarkers were normalized to creatinine. Values in red were deemed to be statistically significant (*p* < 0.05). Covariates adjusted for the models were age, smoking status, and welding duration

Figure 5 presents associations between biomarkers and multiple lung function parameters. A decrease of 0.1 µg/L in plasma 8-isoprostane was associated with a 4.782% decline in FEV_1_ (95% CI 1.540–8.023%, *p* < 0.05), a 6.317% reduction in FVC (95% CI 3.328–9.306%, *p* < 0.05), a 3.719% decrease in PEF (95% CI 0.210–7.229%, *p* < 0.05), and a 9.215% reduction in FEF_75%_ (95% CI 1.851–16.678%, *p* < 0.05). Additionally, a 1 µg/g increase in ∆ α1-antitrypsin correlated with a 0.008% decline in the FEV_1_/FVC ratio (95% CI 0.003–0.013%, *p* < 0.05), while a similar increase in ∆ ITIH4 resulted in a 0.020% reduction (95% CI 0.005–0.035%, *p* < 0.05). A 1 µg/g increase in ∆ 8-isoprostane was linked to a 1.538% decrease in FVC (95% CI 0.126–2.949%, *p* < 0.05). Further, a 1 µg/g increase in post-urinary α1-antitrypsin was associated with a 0.068% decrease in FEV_1_ (95% CI 0.009–0.127%, *p* < 0.05) and a 0.060% reduction in FVC (95% CI 0.003–0.117%, *p* < 0.05). Finally, a 1 µg/g increase in post-urinary 8-isoprostane corresponded to a 5.227% decline in FEV_1_ (95% CI 1.034–9.420%, *p* < 0.05), a 6.686% reduction in FVC (95% CI 2.770–10.603%, *p* < 0.05), and a 4.955% decrease in FEF_25%_ (95% CI 0.540–9.371%, *p* < 0.05).

## Discussion

The novelty of our study is that two potential markers in urine including α1-antitrypsin and ITIH4, could indicate exposure to metal fumes in shipyard workers. These markers were linked to declining lung function. The significant findings of this study are as follows: (1) increasing PM_2.5_ was associated with a decrease in lung function and with increases in urinary α1-antitrypsin and ITIH4, (2) increasing urinary metals were associated with increases in urinary α1-antitrypsin and ITIH4, and (3) increasing urinary α1-antitrypsin and ITIH4 were associated with lung function declines.

Welding processes produce a significant amount of welding fume particles, which are important occupational health concerns. PM_2.5_ was the most common form of suspended welding fume particles in the environment (Chuang et al. 2018; Lai et al. 2016). We conducted this study to determine the level of personal exposure to PM_2.5_ among welding workers in a shipyard. First, the present study found that personal PM_2.5_ exposures ranged 309–464 µg/m^3^ among subjects. In a previous longitudinal study, welding workers were exposed to welding fume PM_2.5_ of 716 µg/m^3^, while office workers were exposed to 114 µg/m^3^ (Lai et al. 2021). According to the United States Occupational Safety and Health Administration (OSHA), our subjects were exposed to less metal fume PM_2.5_ than the occupational exposure limit (OEL) for particulate matter (5 mg/m^3^ for the respirable fraction PM_4_) which is not specifically regulated (Fine et al. 1997). Fe, Zn, and Cu were highly concentrated in the urine of subjects after exposure to welding fume PM_2.5_, which was associated with metals in the welding fume PM_2.5_ (Lai et al. 2016). However, the long-term effects of exposure to relatively lower PM_2.5_ than the OSHA-OEL are still limited.

Lung function declines have been linked to welding fume PM_2.5_. Our previous study showed that this association is potentially attributed to the small size and specific chemical characteristics of these particles (Tung et al. 2022). We found that exposure to welding fume PM_2.5_ was associated with declining lung function among shipyard workers, which is in line with our previous report on welding workers (Tung et al. 2022). This can be explained by welding fume PM_2.5_ causing inflammation and oxidative stress in the respiratory system (Samulin Erdem et al. 2020) and a fall in the vital respiratory capacity and arterial oxygen partial pressure (Subedi et al. 2019), resulting in a reduction in lung function. Declines in FEV_1_ and PEF could be due to impairment of pulmonary function, whereas declines in FEF_25%_ and FEF_50%_ suggest the presence of inflammation respectively leading to obstruction of small and distal airways (Mu et al. 2022). Together, these findings indicate that exposure to welding fume PM_2.5_ increases the risk of lung function declines.

Over a three-year period, we noticed two trends in workers. First, levels of α1-antitrypsin, ITIH4, and 8-isoprostane in the urine dropped after each check-up. Second, higher exposure to welding fumes led to increased levels of α1-antitrypsin and ITIH4 in the urine. A systematic review of welding fume and lung diseases reported that welding fume PM_2.5_ caused acute or chronic inflammation and oxidative stress in the respiratory system, which may lead to COPD (Riccelli et al. 2020). First, a previous observational study showed that the positive association between PM_2.5_ and urinary α1-antitrypsin levels might be related to COPD (Gökhan and Sema 2019). Increased circulating α1-antitrypsin levels could be used as a clinical marker to predict the clinical course of COPD patients without an α1-antitrypsin deficiency (Takei et al. 2019). Moreover, the elevation in α1-antitrypsin, indicative of heightened proteolytic activity suppression, aligns with the molecular narratives of escalated inflammatory and oxidative stress responses (Da 2002). Second, the observed positive correlation between PM_2.5_ and the acute-phase ITIH4 protein corroborates our prior findings, highlighting ITIH4's relevance in prolonged PM exposure and its potential involvement in the pathogenesis of COPD, a condition driven by inflammation (Chen et al. 2021; Lee et al. 2015). Additionally, recent research indicates ITIH4's significant influence on the health impacts of air pollution in occupational settings (Pacheco et al. 2013). These studies collectively underscore ITIH4's role not just in acute but also in chronic disease processes, especially under continuous environmental exposure. Third, variations in the oxidative stress-related 8-isoprostane might be associated with reactive oxygen species (ROS) attributable to metals in welding fumes (Han et al. 2005). Together, inhalation exposure to welding fume PM_2.5_ leads to increased urinary α1-antitrypsin and ITIH4 among shipyard workers, which could increase the risk of COPD development.

Next, we observed that urinary Zn, Cu, Fe, and Ni were most prevalently identified among the shipyard workers. The findings of this study are consistent with those of our previous longitudinal study on welding workers (Lai et al. 2021) and boilermakers (Kim et al. 2004). This suggests that occupational exposure to welding fumes may increase Zn, Cu, Fe, and Ni in urine samples among shipyard workers. Second, we observed that Mn, Co, Ni, Zn, Cu, Fe, and V were positively associated with urinary α1-antitrypsin and ITIH4, which are considered biomarkers of COPD (Takei et al. 2019). A potential relationship between toxic metals from welding fume exposure and COPD was shown among shipyard workers in Korea (Koh et al. 2015). Previous study on welders showed that urinary Ni and Fe were associated with elevated levels of urinary inflammatory biomarkers among welders (Raulf et al. 2016). Furthermore, Cr, Mn, Co, Ni, Zn, Cu, Fe, and V were positively associated with urinary 8-isoprostane in shipyard workers. This study is in line with a previous study that urinary Cr, Mn, Co, Ni, Zn, Cu, and Fe were positively associated with the oxidative stress biomarker, 8-isoprostane, among welders (Hoffmeyer et al. 2012b). Taken together, increasing urinary metals increase the risk of COPD, inflammatory responses, and oxidative stress among shipyard workers, resulting in elevated urinary α1-antitrypsin, ITIH4, and 8-isoprostane.

We found that higher levels of α1-antitrypsin and ITIH4 in urine correlated with worsening lung function, measured as FEV_1_/FVC, in shipyard workers. Notably, it is commonly accepted that a postbronchodilator FEV_1_/FVC ratio of 0.70 is diagnostic of COPD (Lareau et al. 2019). Firstly, we observed a connection between increased levels of urinary α1-antitrypsin and declines in the FEV_1_/FVC ratio. α1-antitrypsin is primarily known as a protease inhibitor, functioning to neutralize enzymes that break down proteins. Elevated levels of α1-antitrypsin in urine might signify that the body is actively trying to counteract protein-degrading enzymes released due to lung tissue inflammation and damage. This mechanism could explain the associated decline in FEV_1_/FVC ratios, as α1-antitrypsin levels may rise in response to the ongoing deterioration of lung function (Takei et al. 2019). Additionally, the specificity of urinary α1-antitrypsin in capturing immediate exposure effects underscores its utility in occupational health assessments, while plasma α1-antitrypsin, being more stable, reflects long-term, systemic impacts and may be related to α1-antitrypsin deficiency (Cazzola et al. 2020)***.*** Therefore, urinary α1-antitrypsin can be used as a biomarker for deterioration of lung function associated with COPD. Secondly, elevated levels of urinary ITIH4 were also associated with FEV_1_/FVC declines among shipyard workers. ITIH4 is an acute-phase protein that is usually upregulated in response to inflammation. Increased levels of urinary ITIH4 may reflect an acute or chronic inflammatory state in the lungs, possibly exacerbated by exposure to welding fumes. This heightened state of inflammation can lead to airway narrowing and obstruction, which in turn can result in declining FEV_1_/FVC ratios (Lee et al. 2015). Together, urinary α1-antitrypsin and ITIH4 could be potential biomarkers of lung function decline in shipyard workers.

This study has several limitations that should be noted. First, the dynamic employment and worksite reassignments inherent in shipyard professions lead to small sample size and subsequent loss of follow-up. Furthermore, we did not analyze the metal composition in metal fume PM_2.5_, which could offer more specific insights into lung function risks. Additionally, while we assessed the presence of various metals in urinary samples, we did not specifically measure chromium six, a key toxic metal in welding fumes. We also did not account for heavy metal intake from food and drink, potentially confounding our findings on exposure to metal fumes. Excluding liver-related diseases did not affect the outcomes, though other factors like psychological status and kidney function, which were not analyzed, might have influenced our findings. Lastly, we did not consider the potential impact of other diseases that might affect inflammatory markers and lung function. Future research should address these gaps, including the specific measurement of chromium six in urinary metals, for a more comprehensive understanding of the relationship between metal fume exposure and lung function decline.

## Conclusions

Our 3-year study reveals that exposure to higher levels of metal fume PM_2.5_ in shipyards is associated with higher declines in lung function and affects urinary biomarkers such as α1-antitrypsin and ITIH4. Notably, these biomarkers showed a decreasing trend over time, yet increased with higher exposure levels, suggesting a complex exposure–response relationship. Our findings highlight urinary α1-antitrypsin and ITIH4 as key markers for the early detection of lung impairment due to occupational exposure, underscoring the importance of continuous health surveillance in industrial settings. The use of urine for non-invasive sampling enhances the practicality of health monitoring in the workplace.

### Electronic Supplementary Material

Below is the link to the electronic supplementary material.

Supplementary Material 1 (DOCX 12973 KB)

## Data Availability

The datasets used and/or analyzed during the current study are available from the corresponding author upon reasonable request.
