# Supplementary material for: Effects of occupational exposure to metal fume PM2.5 on lung function and biomarkers among shipyard workers: a 3-year prospective cohort study
Source: Int Arch Occup Environ Health. 2024 Mar 13;97(4):401–12. doi: 10.1007/s00420-024-02055-1 (PMC10999385; doi:10.1007/s00420-024-02055-1)
Supplement: Supplementary file 1 — Supplementary Material 1 (DOCX 12973 KB) [file 420_2024_2055_MOESM1_ESM.docx]

**Effects of occupational exposure to metal fume PM_2.5_ on lung function and biomarkers among shipyard workers: a three-year prospective cohort study**

Huan Minh Tran^1,2#^ (MD), Ching-Huang Lai^3#^ (PhD), Wei-Liang Chen^4,5,6^ (MD, PhD), Chung Ching Wang^4,5,6^ (MD, PhD), Che-Wei Liang^3^ (MSc), Chi-Yu Chien^3^ (MSc), Chih-Hong Pan^3,7^ (PhD), Kai-Jen Chuang^8,9^ (PhD), Hsiao-Chi Chuang^10,11,12*^ (PhD)

^1^Ph.D. Program in Global Health and Health Security, College of Public Health, Taipei Medical University, Taipei, Taiwan

^2^Faculty of Public Health, Da Nang University of Medical Technology and Pharmacy, Da Nang, Vietnam

^3^School of Public Health, National Defense Medical Center, Taipei, Taiwan

^4^Division of Family Medicine, Department of Family and Community Medicine, Tri-Service General Hospital, Taipei, Taiwan

^5^Division of Geriatric Medicine, Department of Family and Community Medicine, Tri-Service General Hospital, Taipei, Taiwan

^6^School of Medicine, National Defense Medical Center, Taipei, Taiwan

^7^Institute of Labor, Occupational Safety and Health, Ministry of Labor, New Taipei City, Taiwan

^8^School of Public Health, College of Public Health, Taipei Medical University, Taipei, Taiwan

^9^Department of Public Health, School of Medicine, College of Medicine, Taipei Medical University, Taipei, Taiwan

^10^Division of Pulmonary Medicine, Department of Internal Medicine, Shuang Ho Hospital, Taipei Medical University, New Taipei City, Taiwan

^11^School of Respiratory Therapy, College of Medicine, Taipei Medical University, Taipei, Taiwan

^12^Cell Physiology and Molecular Image Research Center, Wan Fang Hospital, Taipei Medical University, Taipei, Taiwan

**Running head:** Biomarkers for lung function decline by metal fume

**^#^These authors contributed equally to this work**

***Corresponding Author**

*Hsiao-Chi Chuang, PhD*

Inhalation Toxicology Research Lab (ITRL), School of Respiratory Therapy, College of Medicine, Taipei Medical University, 250 Wuxing Street, Taipei 11031, Taiwan.

Telephone: +886-2-27361661 ext. 3513. Fax: +886-2-27391143. E-mail: [chuanghc@tmu.edu.tw](mailto:chuanghc@tmu.edu.tw)

**Statements and Declarations**

**Ethics approval and consent to participate**

The study protocol was approved by the Joint Institutional Review Board of Tri-Service General Hospital Ethics Committee (IRB no. 1-102-05-013). It was conducted in accordance with guidelines that were approved. Informed consent was obtained from all subjects before inclusion in the study.

**Consent for publication**

Our manuscript does not contain data from any individual person, so it is “Not applicable.”

**Data availability**

The datasets used and/or analyzed during the current study are available from the corresponding author on reasonable request.

**Competing interests**

The authors declare that they have no known competing financial interests or personal relationships that could have appeared to influence the work reported in this paper.

**Funding**

This study was supported by the Ministry of Science and Technology of Taiwan (111-2314-B-038-079 and 107-2314-B-016 -045 -MY3).

**Authors’ contributions**

Huan Minh Tran and Hsiao-Chi Chuang contributed to the completion of the interpretation of the data and the manuscript. Hsiao-Chi Chuang and Ching-Huang Lai planned the work and designed the experiments. Wei-Liang Chen, Chung Ching Wang, Che-Wei Liang, and Chi-Yu Chien recruited the study cohort and performed personal monitoring. Chih-Hong Pan performed the metal analysis. Hsiao-Chi Chuang performed the biochemical analysis. Kai-Jen Chuang critically revised the manuscript. All authors analyzed and discussed the results and commented on the manuscript.

**Acknowledgments**

Thank you to Ms. Yi-Syuan Lin, Ms. A-Chuan Ho, Ms. Shih-Ting Huang, Ms. Kai-Wei Cheng, and Mr. Huan-Wun Chen for their technical assistance with this study.

**Table S1. Percentage Detection of Metals in Urinary Samples**

| **Urinary Metals** | **Detection (%)** | **<LOD (%)** | **Total (%)** |
| --- | --- | --- | --- |
| **V** | 262 (100) | 0 (0) | 262 (100) |
| **Cr** | 262 (100) | 0 (0) | 262 |
| **Mn** | 262 (100) | 0 (0) | 262 |
| **Fe** | 262 (100) | 0 (0) | 262 |
| **Ni** | 262 (100) | 0 (0) | 262 |
| **Co** | 262 (100) | 0 (0) | 262 |
| **Cu** | 262 (100) | 0 (0) | 262 |
| **Zn** | 262 (100) | 0 (0) | 262 |
| **As** | 180 (68.7) | 82 (31.3) | 262 |
| **Se** | 180 (68.7) | 82 (31.3) | 262 |
| **Cd** | 262 (100) | 0 (0) | 262 |
| **Hg** | 180 (68.7) | 82 (31.3) | 262 |
| **Pb** | 180 (68.7) | 82 (31.3) | 262 |

**Figure Captions**

**Fig. S1** Distribution of particulate matter of ≤ 2.5 µm in aerodynamic diameter (PM_2.5_) and urinary metals among shipyard workers

**Fig. S2** Distribution of plasma and urinary biomarkers among shipyard workers

**Fig. S3** Correlations of 3 biomarkers in plasma, ∆ exposure urine, and post-exposure urine among shipyard workers. Values with an asterisk (*) were statistically significant (p < 0.05)

**Fig. S4** Correlations of 9 metals (µg/g) in post-exposure urine with particulate matter of ≤ 2.5 µm in aerodynamic diameter (PM_2.5_) among shipyard workers. Values with an asterisk (*) were statistically significant (p < 0.05)

**Fig. S5** Associations of post-exposure urinary metals after adjusting for creatinine with lung function in shipyard workers. Values with red color were deemed statistically significant (p < 0.05)

**Fig. S1**

**Fig. S2**


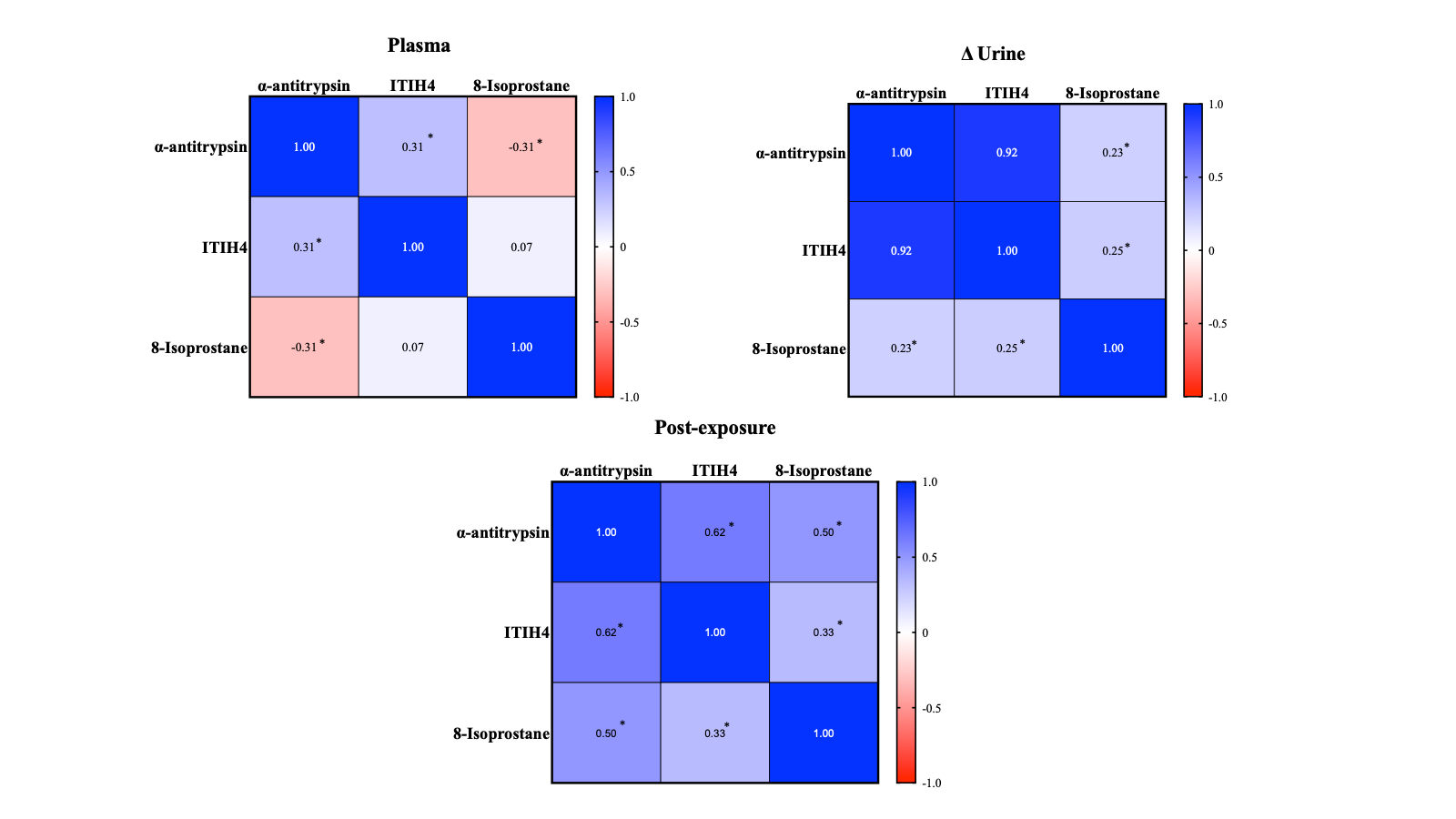


**Fig. S3**


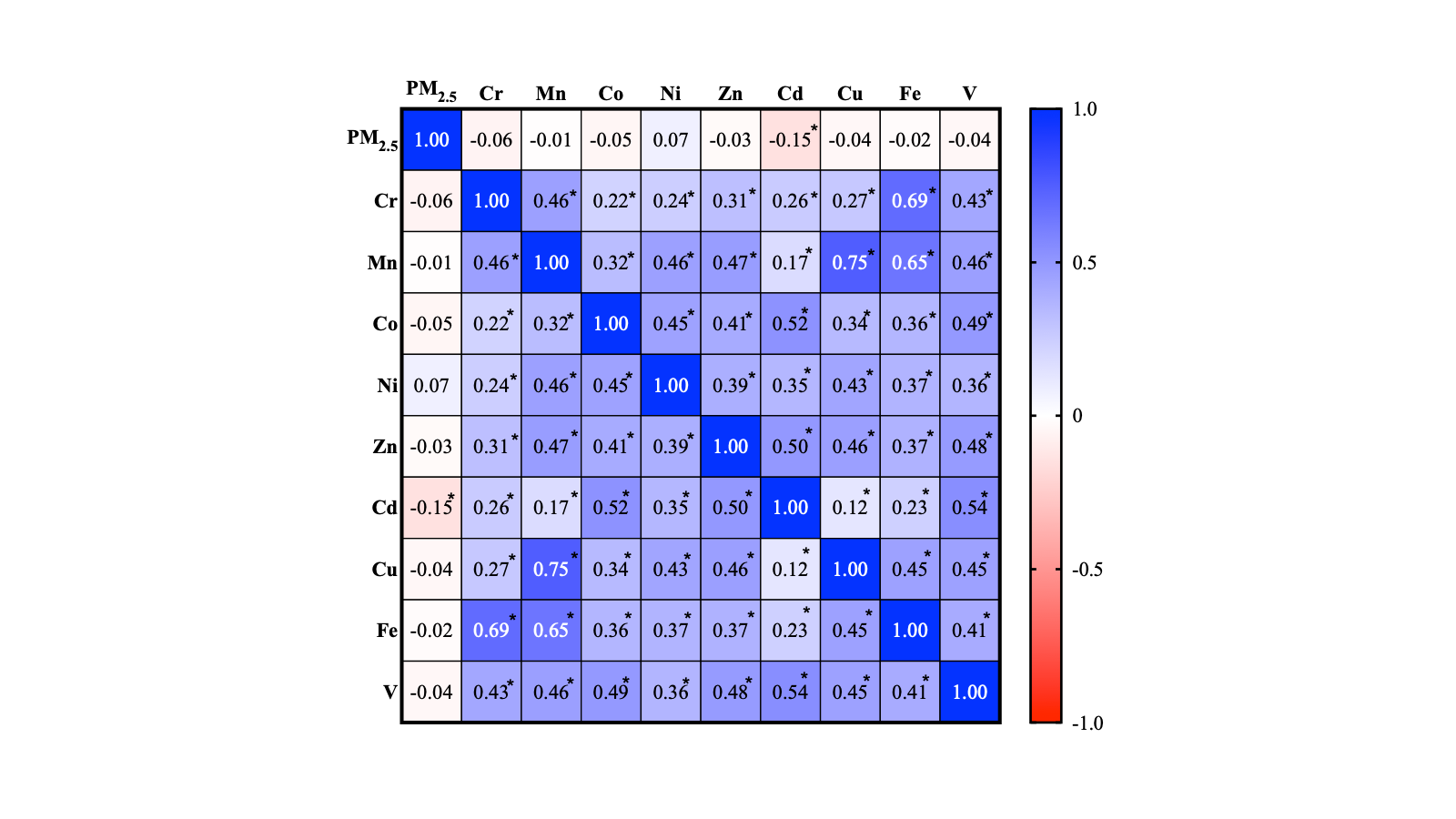


**Fig. S4**


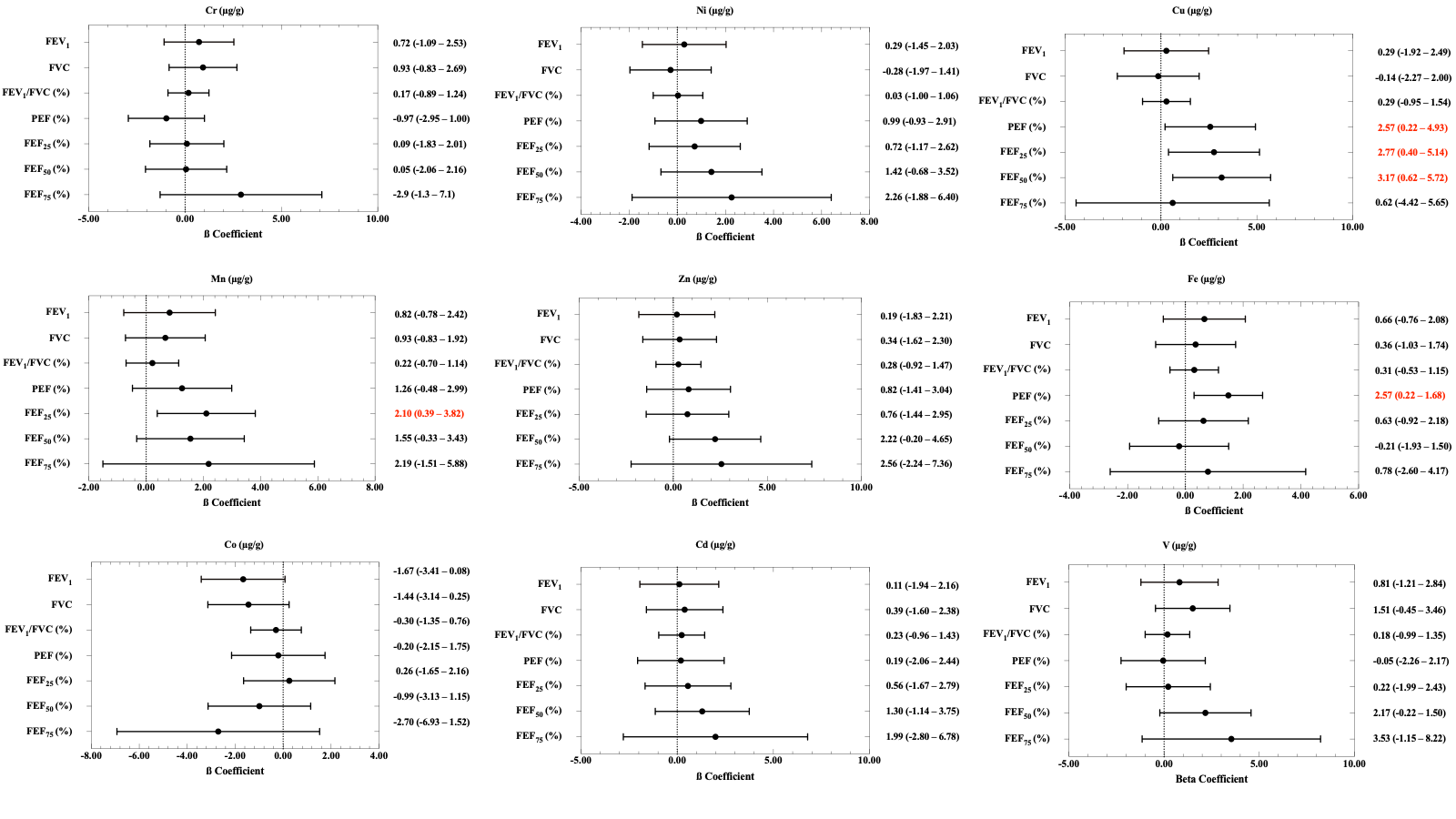


**Fig. S5**
